# Socioeconomic inequalities in dementia risk among a French population-based cohort: quantifying the role of cardiovascular health and vascular events

**DOI:** 10.1007/s10654-021-00788-8

**Published:** 2021-07-25

**Authors:** Noémie Letellier, Sindana D. Ilango, Marion Mortamais, Christophe Tzourio, Audrey Gabelle, Jean-Philippe Empana, Cécilia Samieri, Claudine Berr, Tarik Benmarhnia

**Affiliations:** 1 Institute for Neurosciences of Montpellier INM, Univ Montpellier, INSERM, Montpellier, France; 2 School of Public Health, San Diego State University, San Diego, CA USA; 3 Herbert Wertheim School of Public Health, University of California San Diego, La Jolla, CA USA; 4 Université de Bordeaux, INSERM, Bordeaux Population Health Research Center, UMR 1219, Bordeaux, France; 5 Université de Paris, INSERM U970, Paris Cardiovascular Research Center, Paris, France; 6 Memory Research and Resources Center, Department of Neurology, Montpellier University Hospital Gui de Chauliac, Montpellier, France; 7 Scripps Institution of Oceanography, University of California, San Diego, La Jolla, CA USA

**Keywords:** Cardiovascular health, Dementia, Mediation analysis, Socioeconomic status, Social determinants

## Abstract

This study aimed to investigate the role of cardiovascular health (CVH) and vascular events as potential contributors to socioeconomic inequalities in dementia using causal mediation analyses. We used data from the Three-City Cohort, a French population-based study with 12 years of follow-up, with active search of dementia cases and validated diagnosis. Individual socioeconomic status was assessed using education, occupation and income. A CVH score as defined by the American Heart Association and incident vascular events were considered separately as mediators. We performed multi-level Cox proportional and Aalen additive hazard regression models to estimate the total effects of socioeconomic status on dementia risk. To estimate natural direct and indirect effects through CVH and vascular events, we applied two distinct weighting methods to quantify the role of CVH and vascular events: Inverse Odds Ratio Weighting (IORW) and Marginal Structural Models (MSM) respectively. Among 5581 participants, the risk of dementia was higher among participants with primary education (HR 1.60, 95%CI 1.44–1.78), blue-collar workers (HR 1.62, 95%CI 1.43–1.84) and with lower income (HR 1.23, 95%CI 1.09–1.29). Using additive models, 571 (95% CI 288–782) and 634 (95% CI 246–1020) additional cases of dementia per 100 000 person and year were estimated for primary education and blue-collar occupation, respectively. Using IORW, the CVH score mediate the relationship between education or income, and dementia (proportion mediated 17% and 26%, respectively). Yet, considering vascular events as mediator, MSM generated indirect effects that were smaller and more imprecise. Socioeconomic inequalities in dementia risk were observed but marginally explained by CVH or vascular events mediators.

**Supplementary Information:**

The online version contains supplementary material available at 10.1007/s10654-021-00788-8.

## Introduction

Low socioeconomic status (SES), measured through education, occupation or income, is an important determinant of various health outcomes [1] including premature mortality [2]. The inequalities in health related to SES have been rising in the European Union and in most countries, and one of the major challenges is to understand their modifiable mechanisms to reduce inequalities through equitable interventions [3, 4].

Low SES has been associated with late-life cognitive impairment [5], higher risk of dementia [6], Alzheimer’s Disease [7], and dementia-related death [8]. In the Three-City (3C) Study, a large French cohort, SES was associated with risk of dementia [9] and premature death [10]. The impact of SES on cognitive ageing exists at different life-course periods, from childhood to older ages. In France, a socioeconomic gradient in cognitive impairment was highlighted from middle-aged [11]. Recently, associations between life-course SES and late-life memory function and decline were underlined [5, 12, 13]. Moreover, early life conditions could have an effect on structural brain development, an association between childhood SES and hippocampal volumes in late life was highlighted [14].

Understanding mediating pathways can improve our understanding of the underlying mechanism through which SES influences risk of dementia. SES-dementia relationship can be partly explained by modifiable health conditions and lifestyle factors [15], and identifying and quantifying the role of actionable mechanisms is critical given the lack of existing treatments potentially altering the clinical course of dementia [16] in an aging population.

In this context, cardiovascular health (CVH—a 7-item tool from the American Heart Association AHA including smoking, body mass index, physical activity, diet, total cholesterol, blood pressure, fasting glucose) and cardiovascular diseases (CVD) could be relevant mediators in the SES-dementia relationship. SES inequalities were consistently reported in studies of CVH [17] and CVD [18–20]. Furthermore, cardio-metabolic risk factors (high blood pressure, dyslipidemia, obesity, diabetes) have been associated with early alterations of brain structure and metabolism [21–23] and are risk factors for cognitive decline and dementia [24]. In the 3C Study, ideal CVH was associated with decreased risk of dementia [25]. Conversely, incident coronary heart disease and stroke were associated with an increased risk of dementia [26, 27].

To our knowledge, only two studies investigated modifiable risk factors as potential mediators in the relationship between SES and dementia. In the Toyama Dementia Survey in Japan, a cross-sectional study, lifestyle-related diseases such as diabetes minimally acted as a mediator between low education attainment and dementia [28]. In the English Longitudinal Study of Ageing (ELSA), a mediation analysis shown that 52% of the dementia risk difference between the highest and lowest wealth tertile was mediated by differences in ‘LIfestyle for BRAin health’ (LIBRA) score [29].

Causal mediation analyses can provide a better understanding of complex relationships quantifying the importance of each pathway and thereby allowing to identify potential prevention targets to successful ageing. The objective of this study was to quantify the contribution of CVH and vascular events in the socioeconomic inequalities in dementia risk over 12 years, in a large French cohort. We employed a causal mediation framework and used different decomposition approaches including weighting methods with inverse odds ratio weighting and marginal structural models.

## Methods

### Study population

We used data from the 3C Study, a cohort of 9294 noninstitutionalized participants aged 65 or over enrolled from the electoral rolls of three French cities (Bordeaux, Dijon and Montpellier) in 1999–2001. The main objective of the 3C Study was to assess the risk of dementia and cognitive impairment related to vascular factors [30]. Each participant signed an informed consent. The study protocol was approved by The Ethics Committees of the Hospital of Kremlin-Bicêtre and Sud-Méditerranée III.

We restricted the population to participants with results of a baseline blood draw or data from the questionnaire, without prevalent dementia or history of cardiovascular diseases at baseline, with follow-up for dementia, and without missing information on SES and incident vascular events (Figure S1).

### Socioeconomic status (SES)

Individual SES was evaluated by level of education (primary/secondary and higher [secondary, high school and university], occupational category (blue collars/white collars) and monthly household income (based on the categories of the questionnaire: < 1500€/ ≥ 1500€, i.e. $1800).

### Ascertainment of cardiovascular health, coronary heart disease and stroke

The CVH score, evaluating the level of CVH at baseline, was calculated previously [25] according to the following 7-item proposed by the AHA [31]: smoking, physical activity, healthy diet, body mass index, total cholesterol, blood pressure, and fasting plasma glucose. It was calculated by assigning 0 point for each metric at poor level, 1 point for each metric at intermediate level, and 2 points for each metric at recommended optimal level (total score range, 0–14). For our mediation analyses, CVH score was used as a continuous variable, and each previous item was assessed individually.

The survey of Coronary Heart Disease (CHD) and stroke occurrence during follow-up has been described previously [32, 33]. All suspected incident cases of CHD and stroke were documented by two independent expert committees. CHD included a definite hospitalized angina, hospitalized myocardial infarction, coronary balloon dilatation, arterial bypass or CHD death. Stroke was defined according to the criterion of the World Health Organization. Our analysis was restricted to non-fatal incident CHD and stroke events until the year 2010 (the last visit with validation of vascular events in the cohort).

### Diagnosis of dementia

At baseline and at each follow-up visit, cognitive function was assessed by a trained psychologist using neuropsychological tests (Mini-Mental State Examination, the Isaacs Set Test and the Benton Visual Retention Test). At each follow-up visit, based on neuropsychological performances, neurologists examined participants with suspected dementia to establish a provisional diagnosis [30]. Diagnosis of dementia was then reviewed and validated by an independent committee of neurologists according to the *DSM-IV* criteria. For the present analyses, we considered all incident cases of all-cause dementia over the 12-year follow-up.

### Statistical analyses

The baseline characteristics of study population were described with median (interquartile range) and frequencies (%). To estimate the Total Effect (TE) of SES on incident dementia, we fit a Cox proportional hazards model and an Aalen additive hazards model for each of the three SES indicators with age as the time scale [34, 35].

#### Accounting for selective entry into sample analyzed and censoring during follow-up

Participants excluded from the analytical sample at baseline were older, more often men, with a lower level of education, a lower income, more often blue-collar workers and living in poorer neighborhoods. To minimize the possibility of selection bias, we estimated the inverse of the probability of an individual being censored at baseline using logistic regression, conditional on baseline covariates (age at baseline, sex, centre, education level, APOE genotype, alcohol intake, smoking habits, diabetes, history of vascular pathology, history of respiratory pathology, and neighborhood characteristics). In addition, we calculated an inverse probability of censoring weights (IPCW) to consider informed attrition (including death as a competing event, withdrawal, and lost to follow-up). Each participant was assigned a weight to consider participants who are underrepresented in the analytical data (see Figure S2 and Figure S3). For all analyses, we thus used inverse probability of censoring weighted models to consider both potential differential informed censoring at baseline and during follow-up [36].

#### Causal mediation analyses

To identify if CVH score or incident vascular events (CHD and stroke) were an intermediate between SES-dementia relationship, we conducted causal mediation analyses using the potential outcome framework considering each SES indicator (education, income and occupation) separately [37].

We decomposed the total effect of SES-dementia inequalities into direct effect and indirect effect through CVH or vascular events. We let X_i_ be the SES exposure *i* (education, income or occupation), M_n_ the potential mediators *n* (CVH or incident vascular events), and Y the outcome (incident dementia). In this context, we interpreted the total effect (TE) as the amplitude of SES inequalities in dementia, both directly and through cardiovascular intermediates. The natural direct effect (NDE) is interpreted as the expected level of SES inequality in dementia if the whole population was assigned the mediator status (i.e., CVH or vascular events) of the highest SES group. The natural indirect effect (NIE) represents the change in dementia risk when a given SES indicator is set to the lowest value (e.g., low education) and the mediator changes (e.g., from yes to no vascular events) to what it would have been for the same contrast in the other SES category (e.g., high education). We also tested for the presence of an interaction between each X_i_ and M_n_. The estimation of natural effects is suited for describe the underlying mechanisms of the observed relationship between SES and dementia [38, 39].

To decompose the TE into the part that is explained by CVH score or vascular events (NIE) and the part that is due to other factors (NDE), we decomposed this effect into its direct and indirect effect by fitting two models: a mediator and an outcome model. We included the following covariates in the models: sex, educational level (when the exposure was not education), apolipoprotein Eε4 (APOEε4) carrier status and study centers. When the exposure was income, we also adjusted for living alone (yes/no). We considered spatial clustering by incorporating census *commune* level (municipality) as a random effect for the intercept. TEs, DEs, and IEs and their 95% confidence intervals were estimated via bootstrapping using 1000 independent replications. We calculated proportions mediated (PM in %) as follows: [HR^NDE^ (HR^NIE^ − 1) / (HR^NDE^ x HR^NIE^ − 1)].

We considered as identification assumptions for the interpretation for TE, NDE and NIE, that there were no unmeasured confounders and no exposure-mediator interaction. We also tested for possible interactions between each SES and gender and APOE genotype.

We used two different decomposition approaches to consider the two mediators of interests separately while accounting for their dependence (i.e., CVH affecting vascular events): Inverse Odds Ratio Weighting (IORW) and Marginal Structural Models (MSM).

First, to study CVH score as mediator and decompose the CVH score into this biological and behavioral components, we used Inverse Odds Ratio Weighting (IORW) [40]. IORW condenses information on the odds ratio between treatment and mediators, conditional on covariates, into a weight. This weight, the inverse exposure-mediator odds ratio given covariates, is then used to estimate natural direct effects via weighted regression analysis. Then, indirect effects are identified by subtracting direct effects from total effects [41]. Such approach is particularly well fitted when multiple mediators are considered simultaneously by capitalizing on the odds ratio's invariance property.

Second, to estimate TE, NDE, NIE of SES through incident vascular events, we used MSM with inverse probability weighting (IPW) as recommended in the presence of multiple mediators that are dependent [42]. Indeed, in our setting, CVH is a precursor of incident vascular events and can thus be considered as confounder between vascular events and dementia that is itself induced by the SES exposures of interest. As traditional mediation analyses typically fail in such settings, we employed a MSM to overcome such limitations. In these analyses (with vascular events as the mediator of interest), we considered CVH as a continuous score in the weight calculation. Analyses were performed with SAS 9.4 and RStudio Version 3.6.0.

## Results

### Study population

Finally, 5581 subjects without dementia and history of vascular disease at baseline and with complete data were included (for a total of 45,396 person-years). Approximately 9% of participants (n = 515) developed dementia during this 12 year-period, corresponding to an annual incidence rate of 11.3/1000 person-years. The median age at enrollment was 73 years and 63% were women.

The mean of the CVH score at baseline was higher among participants without incident vascular events (8.2 [SD 1.8] VS 7.6 [SD 1.9] among those with incident vascular events).

In comparison to people without dementia, people who developed dementia were older and more often women (Table 1). They had poorer cardiovascular health, with a mean (SD) CVH score of 7.8 (2.0) (vs. 8.2 [1.8]) and had higher rate of incident vascular events (11.5 vs. 7).

**Table 1:** Individual characteristics of study population according to dementia status (N = 5581)

Individual characteristics, n (%)	Non-demented	Demented
	(n = 5066)	(n = 515)
Age (years)^a^	72.4 (69.0–76.5)	75.6 (72.0–79.5)
Women	3161 (62.4)	352 (68.3)
Study center		
Bordeaux	902 (17.8)	150 (29.1)
Dijon	2869 (56.6)	247 (48.0)
Montpellier	1295 (25.6)	118 (22.9)
Primary education	1109 (21.9)	163 (31.7)
Lower income	1718 (33.9)	216 (41.9)
Blue-collar workers	797 (15.7)	114 (22.1)
APOEƐ4 carrier	939 (18.5)	138 (26.8)
CVH score^b^	8.2 (1.8)	7.8 (2.0)
Incident vascular events	357 (7.0)	59 (11.5)

*CVH* Cardiovascular Health

^a^median (interquartile range)

^b^mean (SD) [total score range, 0–14]

### Total effect of SES on dementia risk

Participants with primary education level had a greater risk of dementia than those with secondary or higher education level (adjusted HR 1.60, 95% CI 1.44–1.78) (Table 2). The risk of developing dementia was higher among blue-collar than white-collar workers, with an adjusted HR of 1.62 (95% CI 1.43–1.84). Lower income was associated with a higher risk of dementia (adjusted HR 1.23, 95% CI 1.09–1.39). When using additive models, 571 (95% CI 269–873), 634 (95% CI 246–1020) and 221 (95% CI − 79–521) additional cases of dementia per 100 000 person and year were estimated for primary education, blue-collar occupation and lower income respectively (Table 2).

**Table 2:** Total effects of socioeconomic level on dementia risk (Weighted Cox proportional hazards model and Aalen model^a^, N = 5581)

	All-type incident dementia (n = 515) n, (%)	Cox PH model		Aalen model	
		HR	(95% CI)	Estimate	(95% CI)
*Educational level*					
Secondary or higher	352/4309 (8.2)	ref	–		
Primary	163/1272 (12.8)	1.60	(1.44–1.78)	571 × 10^−5^	(269 × 10^−5^–873 × 10^−5^)
*Occupational category*					
White-collar	401/4670 (8.6)	ref	–		
Blue-collar	114/911 (12.5)	1.62	(1.43–1.84)	634 × 10^−5^	(246 × 10^−5^–1020 × 10^−5^)
*Income (n* = *5570)*					
Higher income	299/3647 (8.2)	ref	–		
Lower income	216/1934 (11.2)	1.23	(1.09–1.39)	221 × 10^−5^	(− 79 × 10^−5^–521 × 10^−5^)

*HR* hazard ratio; PH: Proportional Hazard; *CI* confidence interval

^a^Adjusted for age, gender, education (for income and occupation models), living alone (for income models), APOE4 and study centers

### Mediation analyses

The results of mediation analyses considering CVH score as mediator using IORW are reported on Table 3. When decomposing the TE into NDE and NIE for education, we observed a direct effect HR of 1.51 (95% CI 1.22–1.88) and an indirect effect HR of 1.08 (95% CI 1.03–1.14) mediated through CVH score, these resulting to 17% (i.e., PM) of the effect of education on dementia being mediated through CVH. When we decomposed the CVH score into biological and behavioral components, behavioral components appeared to more contribute to socioeconomic inequalities in dementia (e.g., with the alimentation component, we observed an indirect effect HR of 1.04 (95% CI 0.99–1.09)). For occupation, we found a direct effect HR of 1.64 (95% CI 1.24–2.17) and an indirect effect HR of 1.00 (95% CI 0.93–1.08) mediated through CVH score. For income, we observed a direct effect HR of 1.19 (95% CI 0.94–1.51) and an indirect effect HR of 1.05 (95% CI 1.00–1.11) corresponding to a PM of 26%. For occupation and income, we found that none of the CVH score components appeared to contribute appreciably to socioeconomic inequalities in dementia (Table 3).

**Table 3:** Total, direct and indirect effects of socioeconomic level through CVH score in binary and each component (using IORW^a^, N = 5581)

	Total effect		Natural direct effect		Natural indirect effect	
	HR	(95% CI)	HR	(95% CI)	HR	(95% CI)
*Primary education*						
**CVH score**^**b**^	1.62	[1.32–1.99]	1.51	[1.22–1.88]	1.08	[1.03–1.14]
Components of CVH score						
Biological components	1.62	[1.32–1.99]	1.61	[1.31–1.99]	1.01	[1.00–1.02]
Behavioral components	1.62	[1.32–1.99]	1.60	[1.30–1.98]	1.01	[0.99–1.04]
Biological components						
Total cholesterol	1.62	[1.32–1.99]	1.62	[1.32–1.99]	1.00	[0.99–1.01]
Blood pressure	1.62	[1.32–1.99]	1.60	[1.30–1.95]	1.01	[1.00–1.03]
Fasting plasma glucose	1.62	[1.32–1.99]	1.61	[1.31–1.98]	1.01	[1.00–1.02]
Behavioral components						
Smoking	1.62	[1.32–1.99]	1.66	[1.35–2.03]	0.98	[0.94–1.00]
Healthy diet	1.62	[1.32–1.99]	1.56	[1.26–1.94]	1.04	[0.99–1.09]
Physical activity	1.62	[1.32–1.99]	1.63	[1.33–1.99]	1.00	[0.98–1.01]
BMI	1.62	[1.32–1.99]	1.61	[1.31–2.00]	1.01	[0.97–1.05]
*Blue collar workers*						
**CVH score**^**b**^	1.64	[1.25–2.11]	1.64	[1.24–2.17]	1.00	[0.93–1.08]
Components of CVH score						
Biological components	1.64	[1.25–2.11]	1.64	[1.25–2.12]	1.00	[0.99–1.02]
Behavioral components	1.64	[1.25–2.11]	1.64	[1.26–2.12]	1.00	[0.96–1.04]
Biological components						
Total cholesterol	1.64	[1.25–2.11]	1.64	[1.25–2.11]	1.00	[0.98–1.01]
Blood pressure	1.64	[1.25–2.11]	1.63	[1.23–2.09]	1.01	[0.99–1.03]
Fasting plasma glucose	1.64	[1.25–2.11]	1.63	[1.25–2.11]	1.00	[0.99–1.02]
Behavioral components						
Smoking	1.64	[1.25–2.11]	1.66	[1.27–2.15]	0.99	[0.96–1.01]
Healthy diet	1.64	[1.25–2.11]	1.65	[1.23–2.16]	0.99	[0.93–1.06]
Physical activity	1.64	[1.25–2.11]	1.64	[1.26–2.11]	1.00	[0.98–1.01]
BMI	1.64	[1.25–2.11]	1.62	[1.23–2.10]	1.01	[0.96–1.07]
*Lower income (N* = *5570)*						
**CVH score**^**b**^	1.24	[0.99–1.58]	1.19	[0.94–1.51]	1.05	[1.00–1.11]
Components of CVH score						
Biological components	1.24	[0.99–1.58]	1.24	[0.99–1.57]	1.01	[0.99–1.02]
Behavioral components	1.24	[0.99–1.58]	1.25	[1.00–1.60]	0.99	[0.97–1.01]
Biological components						
Total cholesterol	1.24	[0.99–1.58]	1.25	[1.00–1.60]	0.99	[0.97–1.01]
Blood pressure	1.24	[0.99–1.58]	1.22	[0.98–1.56]	1.02	[1.00–1.06]
Fasting plasma glucose	1.24	[0.99–1.58]	1.24	[0.99–1.58]	1.00	[0.99–1.02]
Behavioral components						
Smoking	1.24	[0.99–1.58]	1.24	[0.99–1.58]	1.00	[0.99–1.01]
Healthy diet	1.24	[0.99–1.58]	1.25	[1.00–1.59]	0.99	[0.97–1.01]
Physical activity	1.24	[0.99–1.58]	1.23	[0.99–1.57]	1.01	[0.99–1.03]
BMI	1.24	[0.99–1.58]	1.24	[0.99–1.58]	1.00	[0.98–1.02]

*HR* hazard ratio; *CI* confidence interval

The observed differences in total effects between table 2 and table 3 can be explained by the use of bootstraps for table 3

^a^Adjusted for age, gender, education (for income and occupation models), living alone (for income models), APOE4 and study centers

^b^Global CVH score as a continous variable (total score range, 0–14)

The results of MSM when considering vascular events as mediator are in line with these findings, even if the indirect effects were weaker and less precise for all socioeconomic indicators (Table 4).

**Table 4:** Total, direct and indirect effects of socioeconomic level through non-fatal vascular events (MSM^a^ with inverse probability weighting)

	Total effect	Natural direct effect	Natural indirect effect
	HR (95% CI)	HR (95% CI)	HR (95% CI)
Primary education (N = 5526^b^)	1.37 (1.08–1.73)	1.36 (1.08–1.72)	1.00 (0.88–1.14)
Blue–collar workers (N = 5526^b^)	1.63 (1.25–2.12)	1.64 (1.26–2.14)	1.01 (0.86–1.16)
Lower income (N = 5515^b^)	1.16 (0.92–1.47)	1.16 (0.92–1.47)	1.00 (0.88–1.15)

*HR* hazard ratio; *CI* confidence interval

^a^Adjusted for age, gender, education (for income and occupation models), living alone (for income models), APOE4 and study centers

^b^55 participants excluded due to extreme values for IPW

## Discussion

Using data from the 3C Study, a French population-based cohort of 5581 individuals, we decomposed the total effect of SES on incident dementia into direct and indirect effects through cardiovascular health level (CVH score) and incident vascular events (CHD and stroke). The risk of dementia was higher among participants who were blue-collar workers, with a lower educational level or income. Based on the IORW, we found an indirect effect through CVH and vascular events for two of these socioeconomic measures (17% for education and 26% for occupation). However, we did not identify strong and precise indirect effects of CVH and vascular events using weighting decomposition approaches. Overall, we conclude that CVH and vascular events may play a minor role in explaining SES inequalities in the risk of dementia in this study population.

Conceptualizing SES variables as exposures of interest in epidemiological research under the potential outcomes framework has been shown to be a challenging task given the likely violation of the consistency assumption [43]. Consistency, in causal inference, requires that there be a single version of exposure under study and that this version of exposure, as recorded in the data, be the same version of exposure across exposed individuals. For SES variables such as education or income, it has been shown that such assumption is often violated [44] as many pathways between each SES and health are possible. Instead, in our study we rather conceptualized a specific pathway (i.e., cardiovascular) linking each SES variable of interest to dementia risk and aimed at decomposing specific CVH and vascular events pathways that contribute to explain inequalities in dementia risk. In this context, the decomposition analyses allowed us to isolate a specific pathway and provide some potentially useful etiological insights about the appropriateness of strategies focusing in CVH to potentially reduce dementia SES disparities. For example, lifestyle interventions could be implemented to improve CVH (e.g., reduced obesity, diabetes, hypertension in midlife, physical exercise, smoking cessation) targeting low SES groups in order to reduce dementia related SES inequalities, particularly through healthy diet who seems to have a more important role than the other CVH components to explain education-dementia inequalities.

The total effects of SES variables on dementia incidence are in line with previous studies [5–8]. The SES may act through brain reserve capacity and lifelong mental stimulation or other plausible midlife factors that may be mediators such as health behaviors, living conditions, or access to health care. Moreover, SES may influence brain structure and function [45], for example SES can affect the functional development of the prefrontal regions from early childhood [46]. Thus, social inequalities in cognition may be the result of both complex biological mechanisms and socially constructed determinants. Previously, an indirect effect of lifestyle-related diseases in the relationship between SES and dementia was found in the Toyama Dementia Survey in Japan [28] and the ELSA cohort in English population [29]. In the study by Kay et al. [29], they found a larger proportion mediated than in our study. Such discrepancy may be mostly explained by the difference in the number of dementia cases (only 92 cases representing 3.0% in ELSA), the diagnosis of dementia (base on self-reported measures in ELSA) and different age distribution (mean age: 64.9 years vs 73 years in the 3C Study).

Our findings regarding small indirect effects for CVH and vascular events in SES-dementia inequalities could be related to the characteristics of our cohort. Due to exclusion of prevalent cases of CVD and potentially the most exposed individuals, the mediation may be underestimated. Moreover, our population of interest is relatively old, and we thus focused on potential changes in CVD and CVH at later ages. It is possible that CVD and CVH play a more important role in explaining SES-dementia inequalities when conceptualized in the middle-age period. It would be interesting in future analyses to explore such mechanisms in elderly cohorts with CVH documented in midlife or early aging and possibly identify age thresholds under which CVH and CVD play a more critical role in dementia inequalities. In addition, future studies are recommended to explore other potential underlying mechanisms such as environmental exposure or psychosocial factors (e.g., leisure activities, social interaction).

Our study has some limitations. First, our population sample is recruited in three French urban areas limiting the generalization of our results. There is another limitation regarding the association between vascular events and dementia in the 3C study. Usually, stroke has been shown to follow a social gradient with incidence rising as socioeconomic status decreases. However, in the 3C study, a higher level of household income was associated with a higher risk of ischemic stroke [47]. The selective survival hypothesis may provide a potential explanation. Moreover, in the 3C study, we have the ability to look at types of dementia. It would have been particularly interesting to conduct the mediation analysis only with mixed and vascular dementia cases, but we lacked the power to do this analysis (n = 91 incident cases). Our major strengths include the large population sample with long follow-up and the active search to identify incident dementia cases and vascular events, validated by specific independent committees. We also used different decomposition approaches including weighting methods with IORW and marginal structural models in order to consider the two mediators of interests separately while accounting for their dependence. However, such analyses showed inconsistent and imprecise indirect effects.

## Conclusion

The research on social determinants of health is particularly challenging due to the complexity of the causal pathways and the long time periods during which they play out [3], and this is especially true in dementia research as dementia is a multifactorial disease whose neurodegenerative process begins 20 to 30 years before the onset of the first clinical symptoms. In this context, causal mediation analysis can provide a better understanding of complex relationships quantifying the importance of each pathway and thereby allowing to identify potential prevention targets to successful ageing. Our findings highlighted CVH and vascular events play a limited role as mediator between SES and dementia.

## Supplementary Information

Below is the link to the electronic supplementary material.

Supplementary file1 (DOCX 134 KB)

## Data Availability

Data are available on reasonable request. Anonymized data will be shared by request to the 3C scientific committee.
