# Supplementary material for: Socioeconomic inequalities in dementia risk among a French population-based cohort: quantifying the role of cardiovascular health and vascular events
Source: Eur J Epidemiol. 2021 Jul 25;36(10):1015–23. doi: 10.1007/s10654-021-00788-8 (PMC8542549; doi:10.1007/s10654-021-00788-8)
Supplement: Supplementary file 1 — Supplementary file1 (DOCX 134 KB) [file 10654_2021_788_MOESM1_ESM.docx]

**SUPPLEMENTARY DATA**

**9294** participants in 3-City Study baseline sample

**858** excluded (did not provide blood sample and/or did not complete self-administered questionnaire on leisure and physical activities)

**8436** individuals available for study

**906** had dementia and/or history of cardiovascular disease leading to hospitalization at baseline

**358** missing information on ≥1 cardiovascular health metrics or covariates

**7172**

**546** excluded (no follow-up for incident dementia)

**6626**

**19** missing information on occupation

**359** missing information on income

**6252**

**5895**

1. excluded during the IPW calculation

- **257** missing information for covariates
- **57** with extreme values for IPW

**305** coronary diseases at baseline

**5570** participants included in analysis for income exposure

(**11** missing information on living alone)

**5581** participants included in analysis for education and occupation exposure

Figure S1: Flow chart


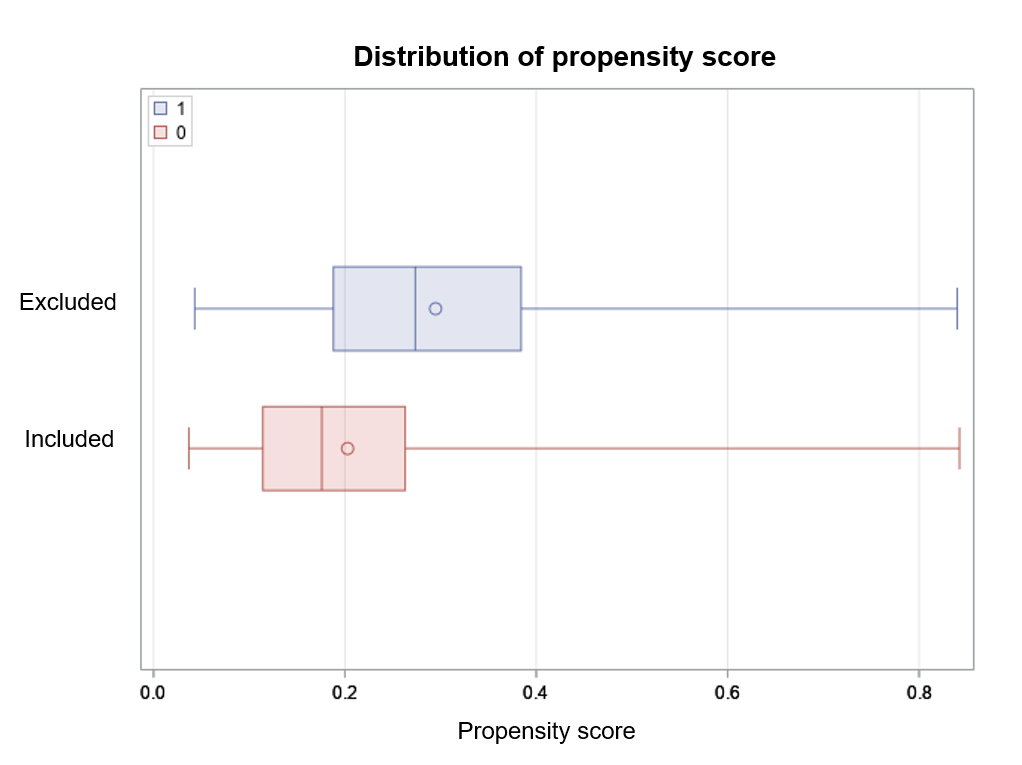


Figure S2. Propensity score distribution of participants excluded or included from the analytical sample at baseline


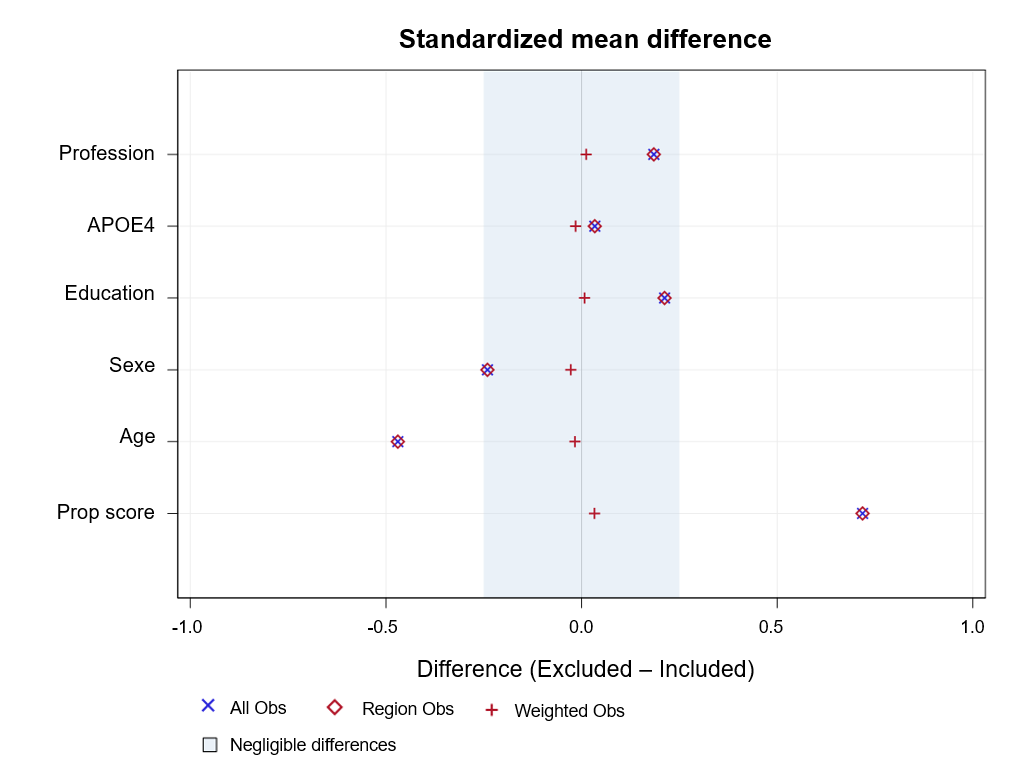


Figure S3. Standardized mean differences
